# Editorial: Molecular mechanisms of thrombosis

**DOI:** 10.3389/fmolb.2024.1497653

**Published:** 2024-09-26

**Authors:** Matteo Becatti, Tom Mckinnon, Claudia Fiorillo, Angelo A. Manfredi, Giacomo Emmi

**Affiliations:** ^1^ Department of Experimental and Clinical Biomedical Sciences “Mario Serio”, University of Firenze, Firenze, Italy; ^2^ Centre of Hematology, Department of Immunology and Inflammation, Imperial College London, London, United Kingdom; ^3^ IRCCS San Raffaele Scientific Institute, Milan, Italy; ^4^ Vita-Salute San Raffaele University, Milan, Italy; ^5^ Department of Medical, Surgery and Health Sciences, University of Trieste, and Clinical Medicine and Rheumatology Unit, Cattinara University Hospital, Trieste, Italy

**Keywords:** thrombosis, molecular mechanism, inflammation, oxidative stress, coagulation

Thrombosis, a leading cause of death worldwide, remains a major challenge in clinical medicine due to its association with serious conditions such as myocardial infarction, stroke, and venous thromboembolism. The incidence of thrombosis rises with age, and its complications are among the leading causes of long-term morbidity and reduced quality of life, especially in Western countries (Wendelboe and Raskob, 2016). Gaining a comprehensive understanding of the pathogenetic mechanisms of thrombosis is crucial for developing effective prophylactic interventions. Despite advancements in understanding its pathophysiology, the molecular mechanisms driving thrombosis are complex and involve a delicate interplay between endothelial cells, erythrocytes, platelets, coagulation factors, and inflammatory mediators (Bettiol et al., 2022; Becatti et al., 2016; Emmi et al., 2021). This Research Topic brings together a collection of studies that explore the intricate molecular pathways contributing to thrombosis, offering new insights that could inform the development of more effective therapeutic strategies.

The study by Wagner et al. employs advanced imaging techniques to visualize clot structures in extracorporeal membrane oxygenation (ECMO) devices, revealing the importance of platelet-leukocyte aggregates and von Willebrand factor in clot stability and dissolution. This work underscores the dynamic nature of thrombus formation, where mechanical forces and cellular components interact to influence clot architecture and stability. Similarly, the research by Yuan et al. delves into the role of Toll-like receptor 4 (TLR4) in thrombus resolution, highlighting the receptor’s involvement in mediating inflammatory responses necessary for thrombus breakdown. In recent years, extensive research has explored the link between inflammation and thrombosis, revealing that the immune and coagulation systems are functionally interconnected (Becatti et al., 2019). The study demonstrates that TLR4 deficiency impairs thrombus resolution in a mouse model, emphasizing the importance of inflammation and immune signaling in the clearance of thrombi. This finding aligns with the broader understanding that thrombosis is not merely a hemostatic event but also an inflammatory process, where immune cells and cytokines play pivotal roles (Stark and Massberg, 2021; Thakur et al., 2023; Morrissey and Smith, 2015; Bettiol et al., 2021).

A review by Khattak et al. further underscores the complex role of platelets in microvascular thrombosis during ST-elevation myocardial infarction (STEMI). The authors discuss how platelets, beyond their hemostatic function, contribute to inflammatory processes and microvascular obstruction, complicating recovery after myocardial infarction. Despite the widespread use of dual antiplatelet therapy, its effect on microvascular thrombosis remains unclear, driving the need for novel therapies targeting specific platelet receptors such as GPVI and PAR4. These emerging treatments show promise in mitigating microvascular damage with a lower risk of bleeding complications, offering new avenues for enhancing therapeutic strategies in STEMI management.

The interplay between coagulation, inflammation and oxidative stress is further explored in the review by Bettiol et al., which examines the regulatory role of SIRT1 in thrombosis. SIRT1, a NAD+-dependent deacetylase, modulates key processes such as endothelial activation, platelet aggregation, and inflammatory response, thereby influencing thrombus formation and stability. This review puts SIRT1 as a potential therapeutic target, suggesting that modulating its activity could offer new avenues for preventing and treating thrombotic diseases.

While these studies focus on the molecular and cellular underpinnings of thrombosis, other contributions in this Research Topic address the clinical implications of these mechanisms. Wu et al.’s multicenter retrospective cohort study evaluates the efficacy and safety of direct oral anticoagulants (DOACs) compared to low-molecular-weight heparin (LMWH) in preventing venous thromboembolism in hospitalized cancer patients. The results suggest that DOACs may offer a safer and more effective alternative to traditional therapies, highlighting the need for personalized treatment approaches based on a deeper understanding of the patient’s thrombotic risk profile and the molecular characteristics of their condition.

Moreover, the impact of novel therapeutic strategies on thrombosis is exemplified in the study by Rodenas et al., which investigates the use of venetoclax, a potent hepsin inhibitor, to reduce the metastatic and prothrombotic phenotypes of colorectal cancer cells. This research bridges the gap between oncology and thrombosis (Fernandes et al., 2019), demonstrating how targeted therapies can simultaneously address tumor progression and thrombotic risk, reflecting the interconnected nature of these pathophysiological processes.

Collectively, the articles published in this Research Topic highlight the complexity of thrombosis as a multifaceted process involving hemostatic, inflammatory, and immune mechanisms. They also emphasize the potential of targeted therapies that go beyond traditional anticoagulation to modulate specific molecular pathways involved in thrombosis. As our knowledge of the molecular mechanisms underlying thrombosis advances, these studies offer a critical foundation for developing more targeted and effective therapeutic strategies, which will ultimately enhance patient outcomes and alleviate the public health burden of thrombotic diseases.
